# Sequential Treatment Based on Sunitinib and Sorafenib in Patients with Metastatic Renal Cell Carcinoma

**DOI:** 10.7759/cureus.4244

**Published:** 2019-03-13

**Authors:** Marcus Schlemmer, Annabel Spek, Severin Rodler, Melanie Schott, Jozefina Casuscelli, Michael Staehler

**Affiliations:** 1 Department of Palliative Care, Barmherzige Brueder Hospital, Munich, DEU; 2 Department of Urology, University Hospital, Ludwig Maximilian University of Munich, Munich, DEU

**Keywords:** metastatic renal cell carcinoma, systemic sequential treatment, target therapy

## Abstract

Objectives: The aim of our study was to evaluate the outcome of alternative sequences of sunitinib followed by sorafenib versus sorafenib followed by sunitinib therapies in patients with metastatic renal cell carcinoma (mRCC).

Materials and Methods: This single-center study analyzed patients with mRCC on systemic therapy between January 2005 and August 2011. Patients were treated with the recommended first-line therapy (sunitinib, sorafenib, pazopanib, or immunotherapy) until progression or intolerable toxicity and afterward switched to another guideline-recommended systemic therapy. Only patients starting first-line therapy on either sorafenib or sunitinib and switching to the other of these drugs were included in this analysis.

Results: Out of 266 patients (females: 85, males: 181) with a median age of 57.1 years (30 - 76 years), 57 patients with a sequence of sunitinib and sorafenib were identified. First-line sorafenib therapy was followed by sunitinib (So-Su) in 32 patients; sunitinib was followed by sorafenib (Su-So) in 25 patients. Progression-free survival (PFS) for patients with first-line sorafenib was 11.6 months and was 8.7 months for sunitinib. Overall survival (OS) rates for Su-So was 118.8 months and 83.3 months with So-Su (p = 0.82). No new safety signals were detected.

Conclusion: None of the therapeutic first-line approaches was superior to the other. Sequencing tyrosine kinase inhibitor (TKI) therapy seems to be effective in mRCC and superior to single-line therapy. Further studies should focus on the efficacy of single treatment lines rather than treatment sequences to estimate more potent drugs based on PFS rather than overall survival (OS).

## Introduction

Renal cell carcinoma is a common cancer in the European Union with approximately 84,400 new cases of renal cell cancer (RCC) and 34,700 kidney cancer-related deaths reported in 2012 [1]. In Europe, mortality rates increased until the early 1990s and stabilized or decreased thereafter. Nevertheless, there were patients with metastatic disease at the time of diagnosis, although a shift to smaller tumours with a good prognosis could be noticed [2]. Life expectancy increased because of improved therapeutic options.

Treatment of metastatic renal cell carcinoma (mRCC) has significantly improved over the past decade with the introduction of targeted therapies. Targeted therapies inhibit the vascular endothelial growth factor receptor (VEGFR) or mammalian target of rapamycin (mTOR) [3]. As the therapeutic efficacy of single agents is limited, it has become the standard of care to employ sequential treatment strategies [4]. There are many studies comparing different therapies and agents, but there is no evidence-based recommendation on how to sequentially apply different medications to optimize the treatment of mRCC patients [4-5]. Choosing the sequence of agents greatly influences patient survival. Guideline-recommended and approved treatment of mRCC in a first-line setting is sunitinib (Su), pazopanib, or bevacizumab, plus interferon alpha (IFN-a), for clear cell RCC; for non-clear cell RCC, the first-line setting is sunitinib, everolimus, or temsirolimus. Sorafenib (So) was the subject of various studies regarding outcome compared to other agents [6-10]. These studies suggested that sorafenib and sunitinib had a clinical benefit when used as first and second-line therapy, one after the other. The first prospective, randomized Phase III study to test the hypothesis that sequential therapy with So-Su was superior to Su-So in prolonging total progression-free survival (PFS) in metastatic RCC was the Phase III Randomized Sequential Open-Label Study to Evaluate the Efficacy and Safety of Sorafenib Followed by Sunitinib Versus Sunitinib Followed by Sorafenib in the Treatment of First-Line Advanced/Metastatic Renal Cell Carcinoma (SWITCH) trial [11].

The subject of this retrospective study was to compare the clinical outcomes of different therapeutic sequential regimens in mRCC patients.

## Materials and methods

Data from a prospective database of mRCC patients treated with systemic therapy between January 2005 and August 2011 at the University Hospital of Munich, Germany were included and analysed.

No pretreated patients were accepted. At the beginning of systemic therapy, 62% of the metastases were localized in the lung, 39% were skeletal metastases, 29% were in the lymph nodes, metastases of the liver in 26%, and brain metastases in 12%. Patients received sunitinib, 50 mg once daily (four weeks on, two weeks off), or sorafenib, 400 mg twice daily. Dose modification to manage side effects was possible. The side effects in our patients did not differ from published experiences [11].

Imaging of patients on systemic therapy was performed using computed tomography (CT) of the chest, abdomen, and pelvis every three months, and brain CT once a year if no brain metastases were known. Response to systemic therapy was categorized according to the Response Evaluation Criteria in Solid Tumors (RECIST) study criteria [12] as complete response (CR), partial response (PR), stable disease (SD), and progressive disease (PD). Patients were treated with a single agent until PD or intolerable toxicity and were subsequently switched to another single agent. PFS and overall survival (OS) were analysed separately for every agent and every therapeutic sequence.

Calculations were performed using the IBM Statistical Package for Social Sciences (SPSS) Statistics for Windows, version 24 (IBM Corp., Armonk, NY, USA). Kaplan Meier statistics was used to analyse the PFS and OS.

## Results

**Figure 1:**
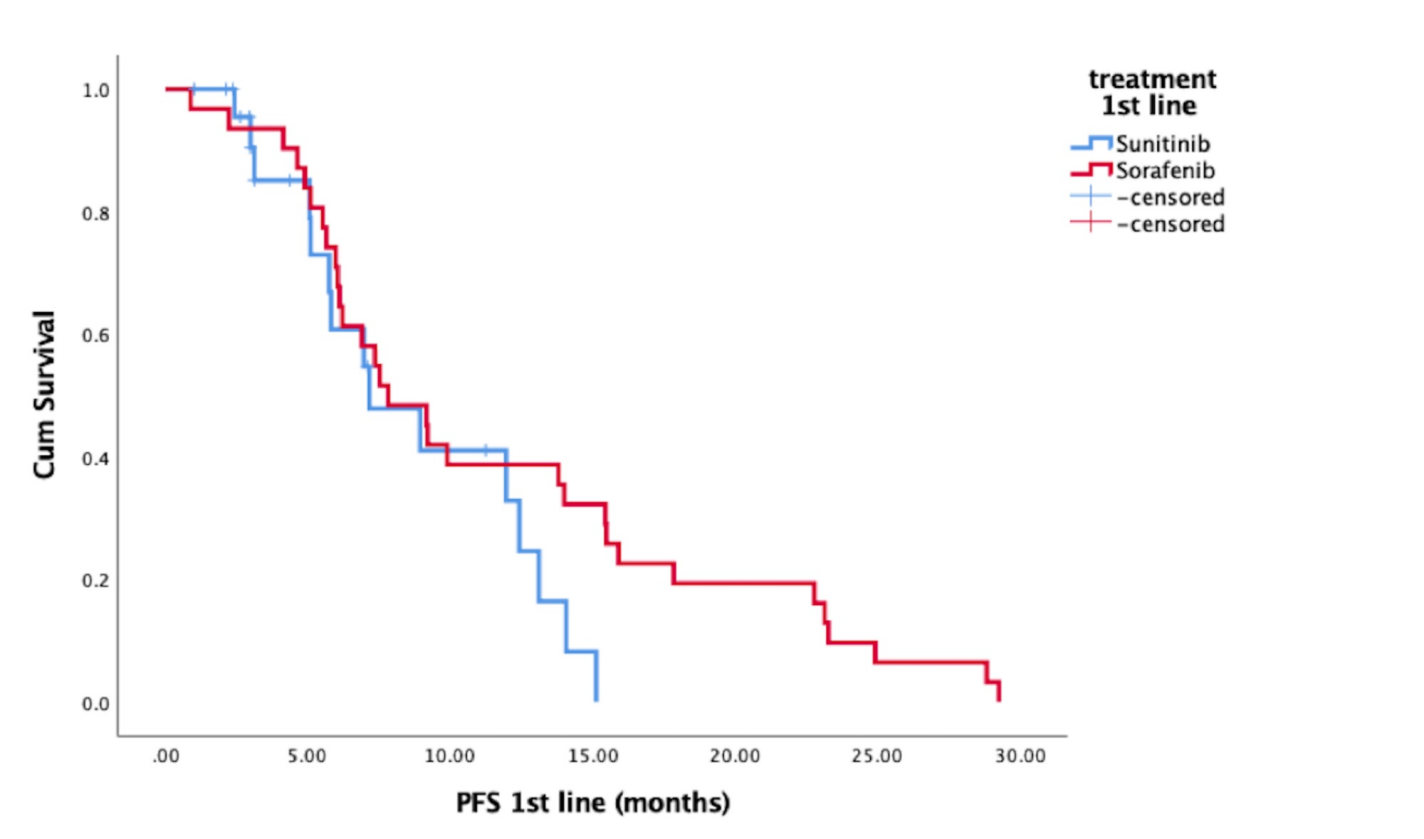
Progression-free survival (PFS) in first-line therapy Cum: cumulative

The median age was 57.1 years (range: 30 - 76 years). The mean number of metastatic sites at the start of systemic therapy was 2.5. Sixty-two percent of the lesions were localized in the lung, 39% were skeletal, 29% had nodal involvement, and 26% had liver lesions, as well as brain metastases in 12%. All patients had a nephrectomy prior to systemic therapy. Patients were classified according to the Memorial Sloan-Kettering prognostic status as low-risk in 25.6%, intermediate in 67.4%, and high-risk in 7.0%. First-line sorafenib therapy was followed by sunitinib (So-Su) in 32 patients; sunitinib was followed by sorafenib (Su-So) in 25 patients. PFS for patients started on first-line sorafenib was 11.6 months (confidence interval (CI): 8.8 - 14.5) and PFS in patients on first-line sunitinib was 8.7 months (CI: 6.7 - 10.8) (not statistically significant) (Figure 1).

**Figure 2:**
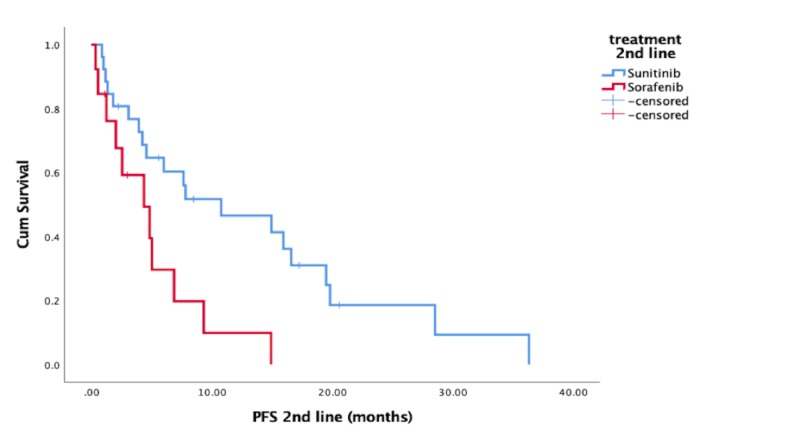
Progression-free survival (PFS) in the second-line therapy Cum: cumulative

Patients on first-line sorafenib and second-line sunitinib had a median PFS of 5.0 months (CI: 2.46 - 7.62) for their second-line drug sunitinib alone. Patients on sunitinib in the second-line therapy after the sorafenib first-line therapy had a significantly longer PFS of 13.8 months (CI: 8.5 - 18.1, p = 0.007) (Figure 2).

Median overall survival (OS) for Su-So was 118.8 months and did not differ significantly from So-Su at 83.3 months (p = 0.82).

No Grade III/IV adverse events, that had not been previously described, were observed.

## Discussion

The treatment algorithm for mRCC has changed completely since the introduction of targeted therapies in 2006 [13]. In oral anticancer therapy, the compliance of patients is important. Drugs not being given intravenously requires patient acceptance to take them on an everyday schedule. Thus, the management of side effects is crucial.

After reporting the antitumor effect in several clinical trials, a total of seven new drugs inhibiting the VEGF pathway or mTOR mechanism were approved a short time thereafter [14]. At this time, therapy with interferon and interleukin-2 was displaced, and systemic therapy with targeted agents became the standard of care. All drugs provided an overall clinical benefit, regardless of the treatment sequence. The aim of this study was to evaluate the efficacy and differences of sequencing targeted therapies for the best clinical and oncological benefit in patients with metastatic renal cell carcinoma.

We retrospectively analysed mRCC patients who were treated at our department from January 2005 until August 2011. No analysis concerning histologic subtypes was done due to the small sample size.

First-line PFS for sorafenib was 11.6 months and was PFS for sunitinib for 8.7 months. Thus, sunitinib and sorafenib were within the previously reported range (4.4 - 11.6 months for sorafenib and 5.1 - 13.1 months for sunitinib) [15-19].

OS for patients starting on sunitinib was 118.8 months versus 83.3 months for patients beginning with sorafenib (without reaching statistical significance). The Treatment Approaches in Renal Cancer Global Evaluation Trial (TARGET) [6] reported an OS of 17.8 months for sorafenib; we documented an OS of 83.3 months. The SWITCH trial could demonstrate that sequential therapy with So-Su was not superior to Su-So [11].

The data and analysis of this study corresponded to the systematic review published by Albiges et al. [20]. In their review, Albiges et al. were able to demonstrate that systemic therapy of mRCC using sequenced tyrosine-kinase inhibitors prolonged survival but no recommendation could be given concerning the optimal sequence strategy. Given our data, we still cannot see that an improved PFS in the first-line setting contributes to an overall survival benefit. It seems to be more important to keep patients on therapy rather than to have the optimal sequence of a specific drug followed by another specific drug. 

We were unable to reveal any parameters to predict PFS or OS in our patient cohort, other than the previously published prognostic criteria (data not shown here). Grade and nodal status especially did not contribute to the effect in contrast to the literature (18, 21-24). In our trial, the subgroups were too small to allow an analysis of different histologic subtypes.

An intriguing finding of our retrospective analysis was the outstandingly long OS in our patients. We can only speculate on the reason for this. One explanation could be that all our patients had a nephrectomy. It is known that in all pivotal trials with anti-angiogenic therapy, nephrectomy is a prognostic factor for response and survival [6, 18, 21-23]. Furthermore, all of our patients included herein were treated with later line therapies as well. We can estimate that the setting of a multidisciplinary tertiary referral center provides more therapeutic options and expertise, translating into longer OS for the individual patient.

As shown recently, OS is not the main driver of patients' decisions in selecting therapies [24]. The benefit of a specific treatment can translate into prolonged PFS but its contributions to an increased OS can neither be proven nor experienced. Thus, the selection of a drug is dependent on multiple factors and PFS is only one of them. In our cohort, we could prove that the second-line efficacy of sunitinib was higher than expected and longer than with sorafenib but did not translate into a significant OS benefit. It remains unclear why the first-line PFS of sorafenib was significantly longer than previously reported, but this could be explained with a lack of alternatives in the reported time frame and a bias based on a need to treat patients beyond progression. This supports the paradigm of treatment beyond progression in sequencing tyrosine kinase inhibitors (TKI) therapy.

Limitations

This study had several limitations. The data were gathered on patients treated between 2005 and 2011 when a significant number of patients had no other treatment options but the two drugs reported on. This leads to a bias not only towards the efficacy data but also the PFS data. PFS was calculated mainly based on the duration of therapy and not necessarily on centrally reviewed RECIST data reporting a real-world setting in unselected patients.

## Conclusions

No general recommendations can be made regarding sequential treatment strategies in patients with mRCC. The principle of sequencing TKI therapy in mRCC is valuable and contributes to the long-term OS. Predictors of follow-up and therapy response should be evaluated in further studies.
